# Diversity, inclusion, and bias in Continuing Medical Education activities: lessons learned from participant evaluations

**DOI:** 10.1080/10872981.2025.2525170

**Published:** 2025-07-04

**Authors:** Melissa D. Bregger, Celia Laird O’Brien, Oluwateniola E. Brown, Linda Suleiman, Sheryl A. Corey, Clara J. Schroedl

**Affiliations:** Feinberg School of Medicine, Northwestern University, Chicago, IL, USA

**Keywords:** Continuing medical education, healthcare professions education, diversity, inclusion, bias

## Abstract

**Purpose:**

Recommendations to ensure diverse, equitable, and inclusive content in Continuing Medical Education (CME) have been developed, however, learners’ perception of these efforts are unknown. Learner recognition of biased or non-inclusive content and satisfaction with activity diversity provides insight into the success of bias mitigation efforts during CME planning and delivery. This study’s objective was to evaluate the types of bias identified by learners, and to evaluate learners’ perception of inclusivity and satisfaction with the diversity of CME activities.

**Study Design:**

This study was a retrospective mixed methods analysis of post-activity evaluation comments from 210 CME activities and 5,284 evaluations at a large Accreditation Council for Continuing Medical Education (ACCME)-accredited academic healthcare system from September 1, 2022 to December 31, 2023.

**Results:**

Learners were satisfied with speaker and content diversity in 98.9% of activities. The qualitative analysis included 967 comments and demonstrated four main categories of perceived bias or lack of diversity identified by the CME activity learners: 1) Bias related to social identity factors, of which racial, ethnic, and gender bias were the most common forms identified by learners; 2) Lack of diversity in speakers, content and delivery; 3) Resistance to bias and inclusion evaluation questions; and 4) Commercial/industry bias. Further, some learners noted the instructional design of certain activities was not inclusive of all learners.

**Conclusion:**

These findings suggest that some CME activity learners perceive various forms of bias and lack of inclusivity and diversity despite efforts to review and mitigate bias in the planning and delivery of CME. While most CME activity learners were satisfied with speaker and content diversity, the data can inform more targeted efforts during the CME planning phase that focus on speaker and content diversity and screening for bias that goes beyond traditional industry/commercial bias.

## Introduction

Continuing Medical Education (CME) is critical for addressing the ongoing educational and professional needs of healthcare professionals and improves physician performance [1]. CME planners have traditionally focused on strategies to mitigate industry bias, but less is known about best practices in planning CME activities to mitigate bias related to identify factors (such as age, gender, race, profession, institution) and to assure that the educational experience is inclusive of all learners. Recommendations for incorporating diversity, equity, and inclusion in CME exist [2,3]. However, whether CME activity learners are satisfied with the diversity of CME activity speakers and content, reasons for any dissatisfaction, and whether they perceive bias is unknown.

While the depth and breadth of content addressed with CME programming is vast, much can be learned by evaluating the experience of CME activity learners. In addition to traditional metrics such as improved knowledge, competence, and performance, understanding learner satisfaction with the diversity of speakers and content provides important insights into whether the educational experience is inclusive for all learners. When bias reporting via an anonymous portal and course evaluations was implemented to elicit feedback of a medical student curriculum, there was increased reporting of bias which allowed for targeted interventions to modify curricula [4]. Likewise, we hypothesized that bias reporting via post-activity participant evaluation might provide a similar formative feedback mechanism in CME activities.

This study had two aims: first, to determine if CME activity learners were satisfied with the diversity of speakers and content and reasons for any dissatisfaction; and second, to determine the types of bias and perception of inclusivity identified by CME activity learners. Lessons learned from evaluating this data could help inform future CME planning efforts to prioritize speaker and content diversity and inclusivity and encourage mitigation of bias that goes beyond traditional commercial/industry bias.

## Methods

We conducted a retrospective mixed methods analysis of CME post-activity evaluation questions at a large ACCME-accredited academic healthcare system occurring between September 1, 2022 and December 31, 2023. The academic healthcare system includes 3 large, urban academic hospitals and 10 smaller community-based hospitals in the Midwest. During the CME application process, course directors attested to reviewing the institutional policy titled, ‘Policy on Inclusive Learning Environment in CME’ and were asked to describe what they have done to ensure the planning and content of the activity is diverse, inclusive and bias-free. All CME activity applications were reviewed and approved by an interprofessional and multidisciplinary CME application review committee.

Beginning September 1, 2022, two questions pertaining to bias, inclusion, and satisfaction with speaker and content diversity were added to our existing CME participant post-activity evaluation: 1)‘Are you satisfied with the diversity of speakers and content of this educational activity? If no, please explain.’ (yes/no multiple choice with optional free response) and 2) ‘Describe any biased content and/or lack of inclusion that you experienced or witnessed as a participant in this activity’ (free response). No definition of ‘bias’ was provided, as we were interested to see how learners interpreted ‘bias’ in the context of CME activities. These questions were modified from the bias reporting questionnaire utilized by our school of medicine and through collaboration with two institutional leaders in diversity and inclusion efforts. The two added evaluation questions were asked to learners attending every accredited live activity or regularly scheduled series (RSS) during our study timeframe, regardless of activity topic. Speakers and learners originated from both home and outside institutions, though further identity or demographic information was not collected for planners, speakers, or learners. Learners attending our CME activities were predominantly physicians (72.2%) with a large minority of nurses, advanced practice providers, trainees, and other professions (27.8%).

We used descriptive statistics to quantify participant satisfaction with speaker and content diversity. We conducted qualitative analyses of participant responses to the two post-activity free response evaluation questions. The unstructured comments were collated in Microsoft Excel and two authors (C.L.O. and C.S.) independently coded the data deductively using the above questions as a structuring framework. This was followed by inductive coding to capture other topics that emerged. The authors met to reach consensus on their findings. All authors reviewed and discussed the categories identified in the analyses.

The Institutional Review Board at Northwestern University deemed this project IRB exempt as it was not human subject research.

## Results

### Quantitative analysis

Post-activity evaluation data were available for 210 accredited CME activities, of which 66 were live activities and 144 RSS. There were 5,284 evaluations included in our analyses (3,131 from live activities and 2,153 from RSS).

Of the 5,284 evaluations, 98.9% of respondents were satisfied with the diversity of speakers and content, with 78.6% of activities noting 100% satisfaction. In total, there were 967 free response comments (878 regarding witnessed/experienced bias, 89 regarding speaker/content diversity satisfaction). Most comments (92.0%) were ‘none’ or ‘non-applicable’; 134 substantive comments were qualitatively analyzed (70 regarding witnessed/experienced bias, 64 regarding speaker/content diversity satisfaction). At least one substantive free response was provided in 25.7% of activities for witnessed/experienced bias and 19.1% of activities for satisfaction with speaker/content diversity.

### Qualitative analysis

CME activity learners reported experiencing or witnessing bias that were mapped to four categories: 1) Bias related to identity factors (10 comments); 2) Lack of diversity in speakers, content and delivery (83 comments); 3) Resistance to bias and inclusion evaluation (13 comments); and 4) Commercial or industry bias (10 comments). These categories were further divided into sub-categories with representative quotations provided (Table 1).

**Table 1.** Qualitative evaluation of free response questions to observed bias and lack of diversity in continuing medical education activities as reported on participant evaluations, September 2022 through December 2023.

Categories and sub-categories	# of Comments	Example Comments
**Bias Related to Identity Factors**		
**Social Identity Bias**	**7**	
*Racial and Ethnic*	*3*	The ‘African American’ daughter was ‘angry’, but the white elderly gentleman in the following case study was ‘frustrated,’ etc.
*Gender*	*2*	Fair number of gender stereotypes perpetuated around ‘personality and characteristics’ of men and women.
*LGBTQ+*	*1*	I also noticed the presenters used heteronormative language to describe parents. All kinds of people can be parents and not all parents are ‘mothers’ and ‘fathers.’
*Generational*	*1*	The first lecture was about the terrible statistics of burn out, but then they had the nerve to complain that millennials and gen z do not want to work as much. Of course we don’t – look at how burnt out the older generations are!
**Professional/Specialty Bias**	**2**	I found the panel’s response to an audience question derogatory. I believe the comment was ‘rogue plastic surgeons.’
**Nonspecific Bias**	**1**	Occasionally when discussing topics impacted by social determinants of health, some of the discussion points included bias, but I think were mostly addressed appropriately.
**Lack of Diversity and Inclusion in Speakers, Content and Delivery**		
**Professional Identity Diversity**	**32**	
*Institutional/Practice setting*	*10*	Would like to see more diversity in institutions/training on the panel discussions.
*Interprofessional*	*10*	Would like more interdisciplinary representation, nursing, therapy, case management and post acute/rehabilitation management.
*Specialty*	*8*	Broader specialty representation would make the experiences more relatable.
*Career stage*	*4*	Most speakers are residents as opposed to experts.
**Social Identity Diversity**	**26**	
*Racial and Ethnic*	*18*	Would like to see people of color as speakers.
*Gender*	*6*	I will say it is interesting and worth noting how often many of the same male faculty members dominate the conversation and commentary, whereas often woman faculty members are not as commonly a part of the conversation.
*Country of origin*	*2*	Immigrants and immigrant health outcomes need to be measured and followed.
**Non-Inclusive Instructional Content and Design**	**14**	
*Content misaligned with learner level*	*6*	There was a lot of medical jargon that nurses do not understand. It needs to be simplified to meet the needs of the audience.
*Lack of interaction/active learning*	*5*	Need greater interactive workshop elements to practice and gain skills.
*Inaccessible technology*	*3*	Some presentations were very difficult to see with visual challenges even sitting close up front.
**Content Addressing Health Inequities**	**7**	I wish the panel also focused on underserved population who do not have access to excellent care.
**Nonspecific Calls for Diversity**	**7**	There could have been a better level of diversity.
**Resistance to Bias and Inclusion Evaluation**	**13**	If by ‘diversity’ you mean the variety to topics and specialists, then ‘yes’ I am satisfied. If by diversity, you mean the sex, gender, race, and age of the speakers, I really don’t care. I just want good speakers and don’t care about race, sex, etc. I am dissatisfied with the stupidity of the question.
**Commercial/Industry Bias**	**10**	The speaker for [course name] was self-promotional and very little evidence-based medicine presented.

Of the reported bias related to social identity, the most frequent forms of bias against individuals/groups observed in CME activities were racial, ethnic, age/generational and gender bias. For example, one learner reported, ‘The African American daughter was “angry”, but the white elderly gentleman in the following case study was “frustrated”.’ Another learner reported ‘The first lecture was about the terrible statistics of burn out, but then they had the nerve to complain that millennials and gen z do not want to work as much.’ Learners who were not satisfied with the diversity of speakers and content in CME activities were most likely to report a lack of professional diversity (e.g., in institutions or disciplines represented), followed by social identity diversity. For example, one learner noted they ‘would like more interdisciplinary representation’ and another learner noted they ‘would like to see people of color as speakers.’ Some learners also noted that the instructional design of activities was not inclusive of all learners. For instance, a few learners noted content that was misaligned with the level of the learner, or that technology was inaccessible. Other comments suggested resistance to efforts to reduce bias and promote inclusion. For example, one learner stated, ‘I just want good speakers and don’t care about race, sex, etc.’ Finally, a small number of learners contextualized bias as industry bias or conflicts of interest. For example, one learner noted ‘The speaker for [course name] was self-promotional and very little evidence-based medicine presented.’

## Discussion

In this study, we found that while learners at CME activities are generally satisfied with the diversity of speakers and content, some learners experienced bias related to protected classes such as race, gender, or age in the CME activities that were offered. Further, learners who were not satisfied with the diversity of speakers and content most frequently cited lack of professional/institutional diversity of speakers or social identity factors of speakers.

Diversity and inclusion in CME are imperative to adequately address the needs of a diverse patient population. CME activities that include perspectives from underrepresented groups improve cultural competency, reduce healthcare disparities, and enhance patient outcomes [5]. Diverse educational content equips healthcare providers with the skills to recognize and address social determinants of health (SDOH) and other factors contributing to inequities in care delivery [6]. Further, attention to speaker institution, profession, specialty, and career stage helps ensure the inclusion of many diverse perspectives and may better address the learning needs of the entire healthcare team. While we are encouraged that most learners at our CME activities were satisfied with the diversity of speakers and content, importantly, we found some who experienced bias and a lack of diversity. Despite the critical importance of diversity and inclusion, CME activities may inadvertently perpetuate bias through content, faculty selection, and delivery methods.

Several strategies could help to address bias and lack of diversity and inclusion in CME. The Accreditation Council for Continuing Medical Education (ACCME) has standards to prevent commercial/industry bias in CME, however, similar requirements do not exist for addressing other forms of bias or diversity [7]. Whether accreditation requirements from the ACCME would lead to meaningful improvements in bias-free and diverse CME is unknown. Short of mandates from the ACCME, individual CME providers must implement local strategies to address bias and diversity. For example, policies detailing expectations for diverse, inclusive, and bias-free CME can be developed and shared with those planning CME activities. Our policy includes strategies to implement during the planning process to foster a diverse and inclusive learning environment and may have contributed to the overall high level of satisfaction with the diversity of speakers and content.

Our findings serve as a reminder that planners and reviewers of CME activities must ensure planners and speakers are truly representative of the target audience and patient population of the activity. Previous studies investigating learner perceived lack of diversity, inclusion or equality in undergraduate medical education have identified many of the same themes as our qualitative analysis, particularly around witnessed racial, ethnic, and gender identity bias [8]. However, perceived professional identify bias may be unique to CME, in part due to the more heterogenous learner population, with more varied health professions and disciplines, institutions, and levels of experience represented in the audience when compared to the learning environment of students or trainees. A lack of diverse planners and speakers can limit the perspectives offered during CME activities. As an additional strategy for improving recognition of the lack of representation that may occur during the planning process, those involved in CME activity planning can review example case scenarios that demonstrate disproportionate representation of patient demographics or neglect the experiences and needs of marginalized groups. This could sharpen their awareness to better assess representation when reviewing CME activities and content. Further, speakers may unintentionally present materials or use language that reinforces stereotypes or excludes certain groups. To address this, speakers can be asked to utilize a regularly reviewed checklist that asks them to consider whether social indicators such as race, gender, or age are discussed in their content, and provides them suggestions for preferred strategies for content delivery [9]. Finally, CME providers should be leaders and adopt best practices when it comes to evaluation and accountability. Establishing metrics within the CME review process to assess bias, diversity, and inclusion, regularly reviewing course content, faculty composition and learner feedback to identify and address areas of improvement should be standard practice. Additional guidelines for recommended best practices to incorporate diversity, equity, and inclusion in CME are provided by the ACCME [2].

Our qualitative analysis revealed three additional important findings. First, some learners demonstrated apathy or resistance to being asked about bias, diversity and inclusion. For example, one learner expressed, ‘If by diversity, you mean the sex, gender, race, and age of the speakers, I really don’t care … ’. Comments like these highlight a common misperception that increasing diversity leads to poorer quality when in fact, data suggest the opposite is true. Indeed, increasing diversity in healthcare and medical education leads to improved patient-provider trust, reduced health disparities, enhanced problem-solving, better educational outcomes, and higher quality of care and patient satisfaction [5,6,10]. CME planners are in a unique position to address this apathy by emphasizing the importance of diversity and inclusion in health professions education and highlighting these improved outcomes. Second, when asked about bias, some learners default to identifying traditional commercial/industry bias rather than other forms of bias that may be present. Given the rigorous oversight by the ACCME and accredited providers to ensure CME content is trustworthy, evidence-based, and non-promotional [7], it is not surprising that some learners equate bias to commercial bias in this context; it is unknown if this is a phenomenon unique to CME compared to other forms of medical education. Third, while infrequent, a few learners identified a lack of inclusion due to inaccessible technology. This serves as an important reminder that activity planners and speakers should ensure content is accessible for all. Evidence-based resources exist to support and encourage best practices in learning design and accessible technology that can be shared with CME activity faculty and planners [11].

Our study has several important limitations. First, our data only represents the experience of a single academic healthcare system. In addition, demographic information for participants and speakers was not known at the time of data collection; as a result, there was no way to assess validity of comments calling out demographic disparities. Moreover, professional identity (i.e., physician vs non-physician) was only available for learners attending live activities (and not RSS), so it is not known to what degree an interprofessional audience led to more perceived bias or lack of diversity. Future work could examine the impact of collecting important identity information on both learners and speakers, to see how these factors might influence perceived bias. Further, our office of CME had a policy on ‘Inclusive Learning Environment in CME’ as well as two mandatory CME application questions focused on how planners will ensure diverse and biased-free content/speakers. As such, our findings may underestimate witnessed or experienced bias or lack of inclusion compared to other institutions without policies or planning interventions aimed to improve diversity, equity, and inclusion in CME. In addition, we only analyzed RSS and live activities; future studies could investigate perceived bias or lack of inclusion and diversity satisfaction in other forms of accredited CME activities. Finally, the generalizability of our approach may be limited in states that limit diversity, equity, and inclusion initiatives or based on national policy. However, the themes we identified could still positively influence activity planning regardless of local or national regulations.

## Conclusion

Learners attending CME activities reported high satisfaction with speaker and content diversity. When reported, racial, ethnic, and gender bias were the most frequent forms of bias against social identities. Learners who were not satisfied with the diversity of speakers and content were most likely to report a lack of professional diversity (e.g., in institutions or disciplines represented), or social identity diversity. These lessons can help inform CME planning efforts to optimize the experience for all learners and ensure all forms of bias are mitigated. Future work will address best practices for addressing these concerns and the creation of a framework for diversity, equity and inclusion in CME.
